# Pharmacovigilance of Herb-Drug Interactions: A Pharmacokinetic Study on the Combined Administration of Tripterygium Glycosides Tablets and Leflunomide Tablets in Rats by LC-MS/MS

**DOI:** 10.3390/ph15080991

**Published:** 2022-08-11

**Authors:** Hamza Boucetta, Wei Wu, Tao Hong, Rui Cheng, Jing Jiang, Chengxi Liu, Min Song, Taijun Hang

**Affiliations:** 1 Key Laboratory of Drug Quality Control and Pharmacovigilance (China Pharmaceutical University), Ministry of Education, Nanjing 210009, China; 2 Department of Pharmaceutical Analysis, China Pharmaceutical University, Nanjing 210009, China

**Keywords:** leflunomide, Tripterygium glycosides tablets, pharmacokinetics, herb–drug interactions, pharmacovigilance, LC-MS/MS

## Abstract

A popular and widely used combination therapy of leflunomide (LEF) and Tripterygium glycosides tablets (TGT_S_) has become a valuable clinical tool in China for the treatment of rheumatoid arthritis. This regimen has not been evaluated either in terms of interaction or toxicity, even given the rising concerns about LEF’s prolonged elimination half-life and TGT’s narrow therapeutic index, in addition to the current trend of using high doses of LEF. Thus, this study determines the potential adverse drug reactions between these two medicines. Reliable validated LC-MS/MS methods were used for the determination of teriflunomide (TER, the only active metabolite of LEF), and the main components of TGT: wilforlide A, wilforgine, wilfortrine, wilfordine, and wilforine. The results obtained from this investigation, as paralleled with the control groups, revealed that the C_max_ and AUC_0-t_ of TER were significantly decreased with separate co-administration, as the C_max_ and AUC_0-t_ were 30.17 ± 1.55 μg/mL and 24.47 ± 2.50 μg/mL, 374.55 ± 15.54 μg h/mL and 336.94 ± 21.19 μg h/mL, respectively (*p* < 0.05). Meanwhile, the pharmacokinetic profiles of the main components of TGT have also been affected by separate co-administration in rats. Therefore, herb–drug interactions between LEF and TGT have been proven.

## 1. Introduction

As an isoxazole derivative with immunomodulatory and anti-inflammatory properties [1], leflunomide (LEF or Arava) is considered as one of the essential FDA-approved disease-modifying anti-rheumatic drugs (DMARDs) for treating active rheumatoid arthritis (RA) [2] in addition to other auto-immune-related disorders, such as psoriatic arthritis [3], systemic lupus erythematosus [3], sarcoidosis [4], and lupus nephritis [4], and it has also been prescribed for renal transplant recipients with chronic allograft dysfunction [5]. In order to inhibit the already autoactivated T- and B-cells’ proliferation, the prodrug of teriflunomide (TER or A77-1726) blocks the function of de novo pyrimidine biosynthesis’ key enzyme dihydroorotate dehydrogenase (DHODH) and tyrosine kinases [6].

**Figure 1:**
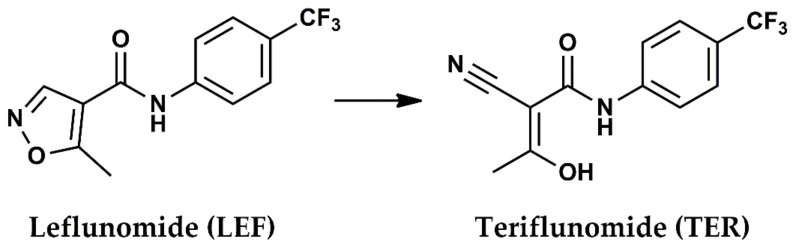
Chemical structure and metabolization of leflunomide (LEF) to teriflunomide (TER).

It is worth mentioning that there is a significant shortage of knowledge regarding the exact mechanisms of action, kinetic profiles, and long-term toxicities of DMARDs including LEF [7]. Additionally, LEF therapy provokes and induces the risk of hepatotoxicity [8]. TER, which represents the active metabolite of LEF (Figure 1), is characterized by the high inter-individual variability of its pharmacokinetic profile [9] and a prolonged elimination half-life [10]. These factors, plus the fact that most of the newly established therapeutic strategies rely on using high doses of LEF for other clinical applications [11,12,13], have given rise to a big concern about the clinical safety of this immunosuppressive, especially with the current tendency of combination therapies [14]. Along with several others, three principal and prominent regimens have been clinically adopted and are based on associating LEF with other agents: (1) immune-suppressive (DMARDs in case of RA), (2) corticosteroids or with traditional Chinese medicines (TCMs) [7].

**Figure 2:**
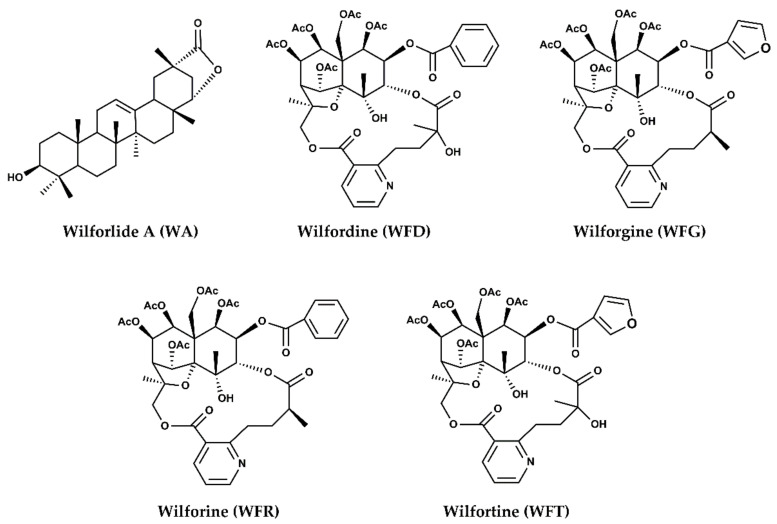
Chemical structure of TGT key components: wilforlide A, wilforgine, wilfortrine, wilfordine, and wilforine.

As a TCM model of these combinations, the active ingredients of *Tripterygium wilfordii* Hook. F. (TwHF) plants—Tripterygium glycosides (TG_s_), which also have distinct anti-inflammatory and immunosuppressive potentialities [15]—have been reported to be associated with LEF [16]. LEF plus Tripterygium glycosides tablets’ (TGT_S_) combination displayed, for instance, remarkable clinical improvements and synergistic effectiveness in treating RA syndrome compared with LEF monotherapy [16]. TGTs have been prescribed for decades (since 1982) in China [17], and ultimately approved by the Chinese National Medical Products Administration (NMPA) for the treatment of inflammation and autoimmune-related diseases [18], such as RA [16], multiple sclerosis [18], nephrotic syndrome [17,18], and following organ transplantation [18]. Sharing almost the same clinical applications with LEF made this herb–drug pair very popular and widely prescribed [17]. TGTs’ multiplicity of activities may be related to the different active ingredients this drug possesses [19], mostly the five major alkaloids (Figure 2): wilforlide A (WA), wilforgine (WFG), wilfortrine (WFT), wilfordine (WFD), and wilforine (WFR) [20]. At the same time, these constituents are also sources of toxicity and probably drug interactions [21].

Furthermore, because of the narrow therapeutic window of this drug [22], its use is limited or at least impacted by dosage optimizations in clinical applications. To the best of our knowledge, no thorough study has tackled the potential herb–drug interaction of LEF (either high or low doses) and TG, notwithstanding all the above-cited factors that may affect the effectiveness or the clinical safety.

The critical nature of evaluating the pharmacokinetic behavior of drugs’ active ingredients is related to different elements. One of these is to maintain their concentrations in the recommended therapeutic index even after combination, especially for those they are intended to treat inflammatory and autoimmune diseases, like RA using LEF and TGT in this case [16]. Additionally, and as mentioned earlier, the ease of reaching toxic levels of TGT constituents, which is related to its very narrow therapeutic index [22], is a likely possibility and can cause toxicity to several organs such as those in the reproductive, hepatic, and renal systems [21]. All these have made this investigation worthy of streamlining combination therapy.

In the present study, relying on a newly developed and validated UHPLC-MS/MS method for quantifying TER (Method I) and on another already developed and validated method in our laboratory [23] for quantifying the amin components of TGT in plasma (Method II), we investigated the potential herb–drug interactions between LEF (high dose) and TGT, based on the pharmacokinetics of TER and TGTs’ main constituents in rats’ plasma after a single oral administration. The acquired results established some scientific basis for future clinical evaluations of the association.

## 2. Results

### 2.1. Method Validation

#### 2.1.1. Selectivity

**Figure 3:**
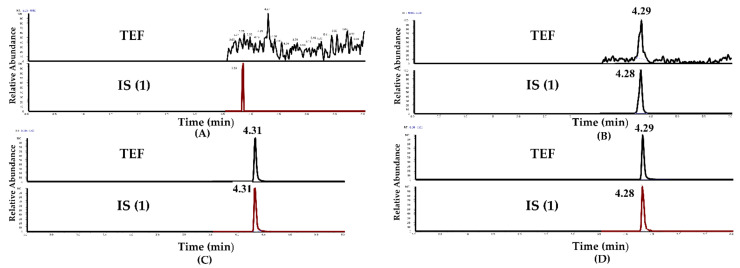
Method (I): representative multiple reaction monitoring (MRM) chromatograms of (**A**) blank rat’s plasma, (**B**) blank rats plasma spiked with TER (1 ng/mL) and IS (1) (1 μg/mL), (**C**) plasma sample from a rat 24 h after a single administration of LEF, and (**D**) plasma sample from a rat 24 h after a co-administration of LEF and TGT.

**Figure 4:**
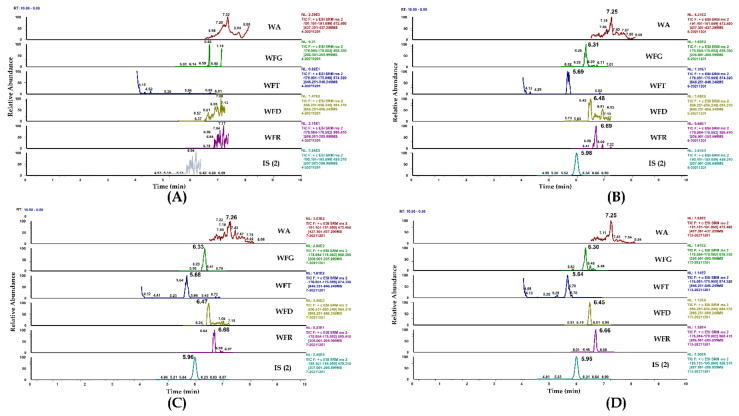
Method (II): representative MRM chromatograms of (**A**) blank plasma; (**B**) blank plasma spiked with WA, WFG, WFT, WFD, WFR (0.05 ng/mL), and IS (2) (200 ng/mL); (**C**) plasma sample from a rat 6 h after a single administration of LEF; (**D**) plasma sample from a rat 6 h after a co-administration of LEF and TGT.

Selectivity was investigated by analyzing blank rat plasma samples from six individuals and comparing them with the corresponding spiked samples. Representative chromatograms of blank rat plasma, blank plasma samples spiked with different analytes and internal standards ISs (Teriflunomide-D4/IS (1) and Irbesartan/IS (2)), and plasma samples after individual and combined oral administrations of LEF and TGT are shown in Figure 3 and Figure 4, respectively.

**Figure 5:**
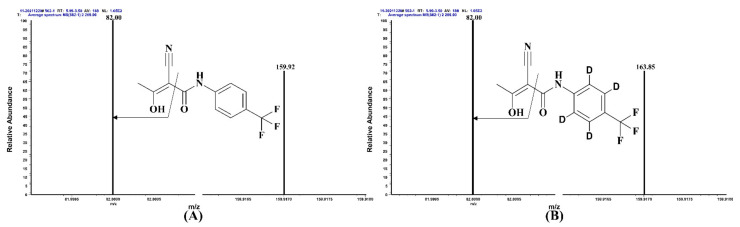
The product ion spectra and chemical structures of TER (**A**) and TER-D4 (**B**).

The results indicated no interfering peaks in the regions of the analyte and ISs, and the methods showed high selectivity. Figure 5 and Supplementary Materials Table S2 display Method (I) and Method (II) product ion spectra after optimization, respectively.

#### 2.1.2. Linearity and Lower Limit of Quantification (LLOQ)

All the calibration curves were linear over the concentration ranges with correlation coefficients better than 0.99. The LLOQ values sufficient for the blood pharmacokinetics study were 1 ng/mL for TER, 0.05 ng/mL for WA, and 0.02 ng/mL for WFG, WFT, WFD, and WFR, respectively, as shown in Table 1 and Supplementary Materials Figures S1 and S2.

#### 2.1.3. Precision and Accuracy

The intra- and inter-batches’ accuracy and precision data for Method (I) are shown in Table 2. The accuracy and precision in the present assay were within the acceptable range set by the ICH for bioassays, indicating that the established method was accurate and precise.

Data related to Method (II) have been included in our previous publication [23].

#### 2.1.4. Extraction Recovery and Matrix Effect

The recovery and matrix effect of TER at LLOQ, low, medium, and high quality concentrations are shown in Table 3. The data are represented by the mean ± standard deviation (mean ± SD). The results indicate that the method’s recovery was high, and the matrix effect was insignificant.

Data related to Method (II) have been included in our previous publication [23].

#### 2.1.5. Stability

The stability of TER in rat plasma is shown in Table 4 and indicates that this analyte is stable at room temperature for 4 h, after three freeze–thaw cycles, in the autosampler at 4 °C for at least 24 h post preparation, as well as stored at −80 °C for 30 days.

Data related to the stability of the main components of TGT using Method (II) have been included in our previous publication [23].

### 2.2. Herbal–Drug Interactions Study

Through a comparative pharmacokinetic study on rats with separated co-administration, the potential herb–drug interactions between LEF and TGT were evaluated. Due to the excellent tolerability of LEF [6] and the current trend of using high doses for some other indications related to autoimmune disorders [11,12,13] on the one hand, and to reach TER steady-state plasma concentrations on the other hand, a single high dose (20 mg/kg) of LEF was used in this study. A clinically equivalent dose of 10 mg/kg/day was utilized for rats in this study, according to the human dose regime of 1.5 mg/kg/day TGT [24]. There are no guidelines indicating the time interval and sequence of the separated co-administration. Thus, an average of 10 min was adopted in this study.

**Figure 6:**
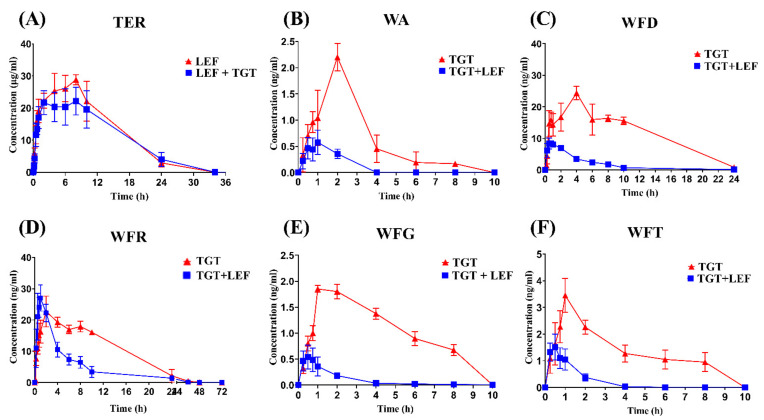
Mean plasma concentration–time curves of TER (**A**) and TGT’s main components: WA (**B**); WFD (**C**); WFR (**D**); WFG (**E**); and WFT (**F**), after the single administration of LEF and TGT, respectively, and their separate co-administration.

The validated LC-MS/MS methods were effectively implemented to determine the concentration of TER, WA, WFD, WFG, WFR, and WFT in rats’ plasma. The mean plasma concentration–time curves of TER and TGT components in rats are shown in Figure 6. The plasma pharmacokinetic parameters are summarized in Table 5.

## 3. Discussion

The obtained results from this investigation, as paralleled by the control group, revealed that TER pharmacokinetic (PK) parameters t_1/2_, C_max_, AUC_0-t_, AUC_0-∞_, and Vd were significantly decreased by 1.46, 1.23, 1.11, 1.11, and 1.32-fold (*p* < 0.05), respectively, unlike the CL, which was significantly increased by 1.5-fold (*p* < 0.05) after the combination.

As for TGT constituents, the plasma level and PK parameters of WA, WFD, WFG, and WFT expressed almost the same types of changes in their profiles after the association, with a significant decrease in t_1/2_, C_max_, T_max_ (except WA, which was not significant), MRT_0-t_, and MRT_0-∞_ (an estimate of 2-fold less; *p* < 0.05), and an approximate of 4-fold minor (*p* < 0.05) of AUC_0-t_ and AUC_0-∞_, while the CL was remarkably augmented for these molecules with an approximate of 4-fold increase (*p* < 0.05) for WA and WFG, and 3-fold (*p* < 0.05) for WFD and WFT. For these four analytes, the Vd has not been altered by the combination.

In contrast, WFR PK profile was slightly different than other TGT ingredients. Our data after the co-administration demonstrated that WFR t_1/2_, T_max_, MRT_0-t_, MRT_0-∞_, and Vd were significantly decreased by 2.4, 1.78, 2.18, 2.45, and 2.70-fold (*p* < 0.05), respectively. In parallel, the C_max_ was found to be considerably increased (2.6-fold; *p* < 0.05). Furthermore, no significant difference in WFR CL rate, AUC_0-t_, and AUC_0-∞_ were noticed.

After absorption, LEF as a prodrug is rapidly metabolized, mainly by the hepatic microsomal enzymes CYP2C9, CYP3A4, and CYP2A1, to TER [25]. TER is a nondialyzable biomolecule, and the hepatobiliary route in unchanged form represents its major elimination way from the body [25]. It has been proven that the PK profile of TER is vulnerable and might be impacted and altered by herbal-based medicines, as Xiao et al. [3] investigated how an ester derivative of paeoniflorin (the major constituent of herbal medicine: *Paeonia lactiflora*); Paeoniflorin-6′-O-benzene sulfonate (CP-25) affects the metabolism, distribution, and excretion of LEF. They have shown that CP-25 increases TER exposure in the synovium and its excretion in urine, feces, and bile; meanwhile, this molecule decreases TER distribution and exposure in the liver, proving the existence of herb–drug interactions and a synergetic effect of associating these two drugs [3].

In this study, LEF’s altered profile (decline of TER C_max_, AUC_0-t_, and AUC_0-∞_) is most likely related to one or more of the above-cited PK stages. In other words, one (or more) of the TGT constituents may have had an effect at one (or more) of these three stages: the distribution, metabolism, and/or elimination of TER. Many studies have proved the ability of WFT and WFR to inhibit CYP3A4 causing drug-induced hepatic injury [26]. Hu et al. [26] evaluated the effect of TGT on PK of losartan and its metabolite EXP3174 in rats, and the findings showed an increase in the plasma concentration of losartan and a decrease of its metabolite, indicating the inhibition of this drug metabolism, and leading also to affirm the herb–drug interactions between these two drugs, although this study has not elucidated the exact mechanism involved in this interaction [26]. Another paper assessed the impact of TG on erythrocyte methotrexate polyglutamates (MTXPGs) in rats, the concomitant administration of MTX and TGT has been largely used in clinical practice to treat RA, and this study came to clarify and rationalize the use. This work proved the safety of the association by revealing the non-affected content of MTX metabolites after the combination [27].

Due to the high hepatoxicity related to TGT-based treatment [26], LEF metabolism might be the point where one or more of TGT ingredients affect by altering the functionality of involved metabolism enzymes, leading to a decrease in TER concentration in blood circulation.

**Figure 7:**
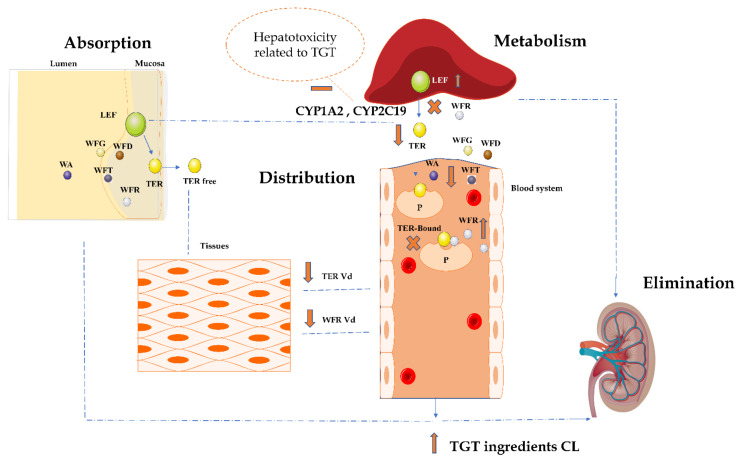
Summary of this work’s findings and TER plus TGT potential stages of interactions’ occurrence.

TER is characterized by a high affinity to albumin in plasma (99% plasma protein-binding) [25], which explains its extended half-life and slow elimination. Our data showed a significant decrease of TER Vd, and this prompts us to assume that one of the TGT ingredients have been involved in competition with TER towards these proteins in plasma. No study has mentioned the affinity of TGT constituents towards plasmatic proteins; therefore, it is not possible to point out precisely the responsible agent for this interaction. However, our results showed that WFR might be intervened and took advantage of the in-plasma pre-existing TER and its high affinity with serum albumin to minimize the WFR binding ratio to express at the end a high concentration (significant increase of C_max_), and so lower the level of Vd without affecting the average exposure (no significant elevation of AUC_0-t_ and AUC_0-∞_). Figure 7 summarizes this work’s findings and TER plus TGT potential stages of interactions’ occurrence. 

This also leads to the presumption that this combination is clinically safe. This conclusion is based on the deliberated study of Gao Xue et al. [28], who considered WFR as in vivo PK and a toxicity marker, which also means clinical safety biomarker of TGT; this came after assessing and proving the dose and time dependency of WFR, unlike WA, WFD, WFG, and WFT [28]. In contrast, all other TGT constituents’ profiles have been affected by showing a low level of concentrations and exposure in plasma (decrease of C_max_, AUC_0-t_, and AUC_0-∞_), leading to the assumption that LEF may promote WA, WFD, WFG, and WFT excretion or metabolism in rats.

## 4. Materials and Methods

### 4.1. Reagents and Materials

Reference standards of Teriflunomide and teriflunomide-D4 (TER-D4) as the internal standard IS (1) were both purchased from Shanghai Yuanyue Bio-Technology Co., Ltd (Shanghai, China). Wilforgine (Lot: wkq20040804), wilfortrine (Lot: wkq20040203), wilfordine (Lot: wkq20040702), wilforine (Lot: wkq20040205), and wilforlide A (Lot: wkq20040901) reference substances (contents > 98%) were all purchased from Sichuan Weikeqi Biological Technology Co., Ltd. (Sichuan, China). Irbesartan (IBs) (Lot: 100607-201804) reference substance (purity > 99%), as another internal standard IS (2), was obtained from the National Institute for Food and Drug Control (Beijing, China).

HPLC-grade methanol and acetonitrile were obtained from Tedia Company Inc., (Fairfield, OH, USA). Analytical-grade ammonium acetate and formic acid were supplied by Nanjing Chemical Reagent Co., Ltd. (Nanjing, China). Purified water was purchased from Hangzhou Wahaha Group Co., Ltd. (Hangzhou, China).

Leflunomide tablets (Lot: 200701, 20 mg) were purchased from Fujian Huitian Biopharmaceutical Co., Ltd. (Fujian, China). Tripterygium glycosides tablets (Lot: 200701, containing 10 mg Tripterygium Wilfordii Hook. F extracts in each tablet) were purchased from Jiangsu Meitong Pharmaceutical Co., Ltd. (Jiangsu, China). The contents of the five major alkaloids in each TGT tablet were determined to be 25.4 µg WA, 86.0 µg WFT, 122 µg WFD, 148 µg WFG, and 175 µg WFR based on our previous quantitative method [2].

### 4.2. UHPLC-MS/MS Conditions

Quantitative analysis of TER (Method I) was conducted with an UHPLC-MS/MS system (TSQ Quantis, Thermo Scientific, San Jose, CA, USA) with an electrospray ionization interface (ESI) operating in negative ion mode. A column of Agilent C18 (2.1 × 50 mm, 1.8 µm) at 40 °C was used for the chromatographic separation. At a flow rate of 0.3 mL/min, 10 mM ammonium acetate buffer solution (0.1% formic acid) and methanol (0.1% formic acid) were set as mobile phases A and B, respectively, in linear gradient elution mode (A:B): 0 min (80:20) → 2 min (80:20) → 3 min (5:95) → 6 min (5:95) → 6.1 min (80:20) → 7 min (80:20). The MS/MS parameters were optimized and set as follows: the spray voltage, 3.5 KV; the ion transfer tube temperature, 350 °C; the vaporizer temperature, 150 °C; nitrogen sheath gas, 241 kPa; and auxiliary gas, 158 kPa. TER quantification was conducted under multiple reaction monitoring (MRM) mode with 0.2 Pa Ar gas collision induced dissociation ion transitions of m/z 269.2 @18 eV → m/z 82.0 and m/z 273.2 @18 eV→ m/z 82.0 for TER and IS (1), respectively.

The chromatographic and quantification analysis technique conditions used for in plasma TGT main components (Supplementary Materials Tables S1 and S2) were the same as those already developed and fully validated in our laboratory [23].

### 4.3. Pharmacokinetic and Herb-Drug Interaction Study Design

Sprague–Dawley rats weighing 180−230 g, aged 8–12, purchased from Shanghai SIPPR-BK laboratory animal Co. Ltd. (Shanghai, China), and housed for 5 days under specific pathogen-free conditions (25 ± 5 °C, 50 ± 20% humidity, 12/12 h light/dark cycle) with free access to food and water were included in the study. All the animal experiments were approved by the Animal Ethics Committee of China Pharmaceutical University (Approval number: 202109007).

Eighteen rats which fasted overnight with free access to water were randomly divided into 3 groups (A, B, and C; *n* = 6). Rats in groups A and C were administrated with a single oral administration of LEF (20 mg/kg) and TGT (10 mg/kg: equivalent to 25.4 μg/kg WA, 148 μg/kg WFG, 86.0 μg/kg WFT, 122 μg/kg WFD, and 175 μg/kg WFR) in 0.5% sodium carboxymethylcellulose (CMC-Na), respectively, while group B rats received a separated co-administration of the same doses (an oral dose of LEF 10 min after an oral dose of TGT).

The blood samples (about 0.2 mL each) were collected from the orbital venous plexus into the heparinized tubes at 0, 0.083, 0.167, 0.25, 0.5, 0.75, 1, 2, 4, 6, 8, 10, 24, 34, 48, and 72 h after the initial administration from each rat. The plasma samples were separated after centrifugation for 10 min at approximately 1100× *g* and stored at −80 °C until analysis.

### 4.4. Sample Preparation

#### 4.4.1. Calibration Standards

Stock solutions of TER (1000 μg/mL), TER-D4 (100 μg/mL), and 1 mg/mL of WA, WFG, WFT, WFD, WFR, and IBs were all separately prepared in methanol. The stock solutions were stored shaded from light at −80 °C, then serially diluted with methanol to produce the required working standard solutions.

Calibration plasma standards were prepared by spiking the appropriate amount of the working standard solutions in blank plasma to yield with the plasma concentrations of 1, 2.5, 5, 10, 25, 50, 100, 250, 500, 1000, 2500, and 5000 ng/mL for TER, and 0.01, 0.02, 0.05, 0.1, 0.2, 0.5, 1, 2.5, 5, 10, 25, 50, and 100 ng/mL for each of the five TGT constituents, respectively.

#### 4.4.2. TER and TGT Constituents

For the quantification of TER, the aliquots of 70 μL plasma were spiked with 10 μL of the ISs solution (1 µg/mL of TER-D4 and 200 ng/mL of IBs in acetonitrile) and mixed with 1 mL acetonitrile to precipitate the protein and extract the analytes; the mixture was vortexed for 5 min and centrifuged at approximately 16,000× *g* at 4 °C for 10 min. A part of the supernatant was collected and diluted 20 times with an acetonitrile–water mixture (60:40, *v*/*v*), then 20 μL of the obtained solution was injected into the UHPLC-MS/MS system for TER analysis using Method (I) parameters and conditions. As for TGT components’ quantification, the other part of the supernatant was evaporated to dryness at 40 °C using a ZLS-1 vacuum centrifugal concentrator. The residue was reconstituted with 100 μL of the methanol/water mixture (60:40, *v*/*v*) and centrifuged again. Finally, a 20 μL aliquot was injected into the UHPLC-MS/MS system using Method (II) parameters and conditions for quantification.

### 4.5. Method Validation

According to ICH guidelines, the LC-MS/MS methods were validated for selectivity, linearity, accuracy, precision, recovery, matrix effects, and stability.

#### 4.5.1. Selectivity

Selectivity was investigated by analyzing blank rat plasma samples from six individuals and comparing them with the corresponding spiked samples, and then after the separate co-administration of LEF and TGT.

#### 4.5.2. Linearity and Lower Limits of Quantification (LLOQ)

The linearity was determined by plotting the area ratios of the analyte to IS (Y) versus the nominal concentrations (X) of each analyte with a least square linear regression (weight coefficient 1/X2). Calibration curves had a total of twelve points and the lowest point was considered as LLOQ, which was used to assess the sensitivity of the six prepared replicates.

#### 4.5.3. Precision and Accuracy

The intra-day and inter-day (*n* = 6) precision and accuracy were evaluated using three different quality control (QC) samples for each analyte. The expression of precision is the relative standard deviation (RSD, %) from the theoretical concentrations.

#### 4.5.4. Extraction Recovery and Matrix Effect

The recovery was assessed by comparing the peak area ratio of each analyte to the ISs obtained from the extracted spiked samples with those of the post-extraction spiked samples. The matrix effect was evaluated by comparing the peak areas of the post-extraction spiked QC samples with those of corresponding standard solutions. These procedures were repeated six times at four QC concentration levels.

#### 4.5.5. Stability

The stability of the analytes was evaluated by analyzing three replicates (*n* = 3) of plasma samples at three concentration levels: low, medium, and high. Samples would experience different conditions: short-term stable storage at room temperature for 4 h, three freeze–thaw cycles at −80°C, post-preparative stability in the autosampler for 24 h, and long-term stable storage at −80 °C for 30 days. All the samples were analyzed, and the acceptance criteria were set at ±15% (RSD% and RE%).

### 4.6. Statistical Analysis

Pharmacokinetic parameters were evaluated with WinNonlin 8.1 (Pharsight, St. Louis, MO, USA). As for statistical significance, it was determined using GraphPad Prism 8.0 (GraphPad Software, Inc., San Diego, CA, USA) via a two-tailed Student’s *t*-test. Statistical differences were considered significant when *p* < 0.05.

## 5. Conclusions

In this study, an efficient and accurate UHPLC-MS/MS method was developed and validated for the determination and traceability of the active metabolite of LEF, i.e., TER, and was successfully applied for the quantitative analysis of TER in rat plasma.

A comparative study of the individual and combined use of LEF and TGT was investigated based on the developed fast and sensitive UHPLC-MS/MS methods used to detect LEF metabolite and TGT active ingredients. Our study proved the existence of drug–herb interactions between these two medicines. The oral administration of TGT has the effect of decreasing TER existence and exposure in plasma. The TGT constituents WA, WFD, WFG, and WFT reacted as well, showing the same changes. The decreased plasma concentrations of all these elements may affect the efficiency of the treatment. The exception was WFR, for which increased plasma concentrations were noticed without having any change to the average exposure overall, ensuring the safety of the combination.

## Figures and Tables

**Table 1:** Linearity ranges, calibration, and LLOQ of TER, WA, WFG, WFT, WFD, and WFR in rat’s plasma.

Analytes	Regression Equation	Linear Range (ng/mL)	R^2^	LLOQ (ng/mL)
TER	Y = 0.0148x + 0.2126	1–5000 ^#^	0.9997	1
WA	Y = 0.004174 X − 0.001699	0.05–100	0.9986	0.05
WFG	Y = 0.01266 X − 0.01096	0.02–100	0.9964	0.02
WFT	Y = 0.008693 X − 0.007188	0.02–100	0.9956	0.02
WFD	Y = 0.01460 X − 0.01277	0.02–100	0.9973	0.02
WFR	Y = 0.003441 X − 0.003744	0.02–100	0.9935	0.02

^#^ The samples presented in a concentration exceeding the range of quantification were diluted with the blank matrix to bring the concentrations into range.

**Table 2:** Precision and accuracy for TER in rat’s plasma.

Analyte	Added Concentration (ng/mL)	Intra-Batch (*n* = 6)		Inter-Batch (*n* = 18)	
		RSD (%)	RE (%)	RSD (%)	RE (%)
	1	4.23	13.57	1.14	12.87
TER	2.5	11.57	−0.16	2.08	5.92
	100	0.89	−0.49	0.27	−0.21
	2500	3.53	3.53	1.35	3.18

**Table 3:** Recovery and matrix effect of TER and IS (1) in rat’s plasma (*n* = 6).

Analyte	Added Concentration (ng/mL)	Recovery		Matrix Effect	
		(Mean ± SD%)	RSD (%)	(Mean ± SD%)	RSD (%)
	1	90.5 ± 4.8	5.3	91.1 ± 6.2	6.82
TER	2.5	96.0 ± 3.8	3.9	100.9 ± 2.7	2.68
	100	98.4 ± 1.1	1.08	98.0 ± 3.6	3.7
	2500	96.1 ± 1.4	1.43	99.7 ± 2.2	2.17
TER-D4 (IS 1)	1000	88.2 ± 3.9	4.42	100.8 ± 4.0	3.96

**Table 4:** Stability of TER in rat’s plasma (*n* = 3).

Analyte	Added Concentration	Room Temperature for 4 h		Autosampler for 24 h (−4 °C)		Three Freeze-Thaw Cycles (3 Times, −80 °C)		−80 °C for 30 d	
	(ng/mL)	RSD (%)	RE (%)	RSD (%)	RE (%)	RSD (%)	RE (%)	RSD (%)	RE (%)
TER	2.5	8.66	11.52	9.24	4.67	6.15	−8.59	3.20	−9.92
	100	9.50	10.56	6.35	−1.19	5.89	−5.63	2.91	−9.96
	2500	1.74	2.57	0.66	−3.66	1.89	−4.99	2.67	−7.83

**Table 5:** Mean plasma concentration–time curves of TER and TGT’s main components after the single administration of LEF and TGT, respectively, and their separate co-administration.

Plasma	TER ^#^		WA		WFD		WFG		WFR		WFT	
	LEF	LEF+TGT	TGT	TGT + LEF	TGT	TGT + LEF	TGT	TGT + LEF	TGT	TGT + LEF	TGT	TGT + LEF
C_max_ (ng/mL)	30.17 ± 1.55	24.47 ± 2.50 ***	1.3 ± 0.3	0.790 ± 0.478 *	12.5 ± 2.01	9.01 ± 0.86 **	1.67 ± 0.78	0.57 ± 0.22 **	10.6 ± 1.52	27.73 ± 3.96 ****	2.97 ± 1.06	1.60 ± 0.41 *
T_max_ (h)	6.67 ± 1.10	6 ± 1.63	1.57 ± 0.53	1.13 ± 0.74	1.43 ± 0.53	0.625 ± 0.137 **	1.57 ± 0.53	0.5 ± 0.2 **	1.86 ± 0.38	1.04 ± 0.51 *	0.96 ± 0.09	0.56 ± 0.12 ****
AUC_0-t_ (ng* h/mL)	374.55 ± 15.54	336.94 ± 21.19 **	4.67 ± 0.26	1.0378 ± 0.34 ****	137 ± 24.08	40.0 ± 4.50 ****	5.6 ± 2.29	1.06 ± 0.31 ***	137 ± 20.5	141.42 ± 23.22	8.64 ± 3.48	2.08 ± 0.75 **
AUC_0-∞_ (ng* h/mL)	374.74 ± 15.49	337.02 ± 21.17 **	4.92 ± 1.01	1.23 ± 0.27 ****	138 ± 25.4	44.85 ± 5.07 ****	6.52 ± 2.74	1.09 ± 0.33 ***	141 ± 21.6	148.07 ± 14.60	8.75 ± 3.49	2.20 ± 0.66 **
t_1/2_ (h)	7.70 ± 0.73	5.26 ± 0.704 ***	2.11± 0.62	1.05 ± 0.0175 **	8.24 ± 1.36	3.96 ± 0.96 ****	3.92 ± 0.57	2.04 ± 0.64 ***	14.4 ± 1.93	6.0 ± 1.21 ****	2.57 ± 1.11	1.08 ± 0.39 *
MRT_0-t_ (h)	9.147 ± 0.909	9.86 ± 1.04	2.99 ± 0.48	1.36 ± 0.23 ****	10.9 ± 2.01	4.43 ± 1.10 ***	3.16 ± 0.18	1.78 ± 0.21 ****	16.1 ± 1.78	7.37 ± 0.62 ****	2.83 ± 0.62	1.09 ± 0.22 ****
MRT_0-∞_ (h)	9.186 ± 0.918	9.88 ± 1.04	3.84 ± 0.75	1.78 ± 0.18 ****	11.2 ± 2.29	5.97 ± 1.27 ***	4.87 ± 0.39	2.08 ± 0.2 9 ****	18.4 ± 2.18	7.48 ± 0.64 ****	3.01 ± 0.59	1.26 ± 0.15 ****
Vd (L/kg)	0.304 ± 0.06417	0.230 ± 0.04967 **	16.3 ± 6.1	23.22 ± 10.9	10.6 ± 1.49	13.81 ± 1.85	155 ± 83.3	268.39 ± 95.0	26.3 ± 5.96	9.72 ± 2.79 ***	39.7 ± 17.2	52.22 ± 8.71
CL (L/h/kg)	0.02 ± 0.0015	0.030 ± 0.0027 *	5.35 ± 1.12	20.57 ± 5.58 ****	0.91 ± 0.11	2.75 ± 0.31 ****	28 ± 15.4	126.19 ± 22.78 ****	1.26 ± 0.19	1.19 ± 0.12	11.6 ± 5.5	31.44 ± 3.85 ****

# For TER, Cmax and AUC0-t/AUC0-∞ are expressed in μg/mL and μg h/mL, respectively. * *p* < 0.05, ** *p* < 0.01, *** *p* < 0.005, **** *p* < 0.0001, significant difference from the TGT and LEF separate co-administration group.

## Data Availability

Data is contained within the article.

## Supplementary Materials

The following supporting information can be downloaded at: https://www.mdpi.com/article/10.3390/ph15080991/s1, Figure S1; Linearity of TER in rats plasma samples; Figure S2; Linearity of WA, WFD, WFR, WFG, and WFT in rats plasma samples; Table S1: HPLC-MS/MS conditions for WA, WFD, WFR, WFG, WFT, and IS (2) determination; Table S2. The optimized parameters / product ions of WA, WFD, WFR, WFG, WFT, and IS (2).
