# Supplementary material for: Pharmacovigilance of Herb-Drug Interactions: A Pharmacokinetic Study on the Combined Administration of Tripterygium Glycosides Tablets and Leflunomide Tablets in Rats by LC-MS/MS
Source: Pharmaceuticals (Basel). 2022 Aug 11;15(8):991. doi: 10.3390/ph15080991 (PMC9412457; doi:10.3390/ph15080991)
Supplement: Supplementary file 1 [file pharmaceuticals-15-00991-s001.zip › pharmaceuticals-1827943-supplementary.pdf]

Supplementary Materials

Table S1. HPLC-MS/MS conditions for WA, WFD, WFR, WFG, WFT, and IS (2) determination.

	**HPLC Conditions**
An UHPLC-MS/MS system (TSQ Quantis, Thermo Scientific, San Jose, CA, USA) equipped with an electrospray ionization interface (ESI) operating in positive ion mode was used for the analysis	
Column	BDS HypersilTM C8 column (150 × 2.1 mm, 2.4 μm)
Column temperature and auto-sampler tray temperature	40°C and 4 °C
Mobile phases	A: Water + 10 mM ammonium acetate buffer solution + 0.1% formic acid<br>B: Methanol + 0.1% formic acid
Linear gradient elution mode (A:B)	0 min (40:60) → 2 min (40:60) → 3 min (0:100) → 9 min (0:100) → 9.1 min (40:60) → 10 min (40:60)
Flow rate	0.5 mL/min
Injection volume	20 μL
	**MS/MS Conditions**
Ion source	ESI
Ion mode	Positive
Optimized mass spectrometry parameters	Spray voltage: +4,500V; Vaporizer temperature: 150℃; Capillary ion transfer tube temperature: 350℃; Sheath gas (N2) pressure: 241 kPa; Collision gas (Ar) pressure: 0.266 Pa; Sweep gas (N2) pressure: 3.4 kPa; Auxiliary gas (N2) pressure: 158 kPa.

Table S2. The optimized parameters / product ions of WA, WFD, WFR, WFG, WFT, and IS (2).

Analytes	[M−H]+ (m/z)	Electrospray ionization (ESI) source	MRM transitions (precursor → product)	Collision energy (eV)
WA	472.46	ESI (+) ionization	472.46→437.30	12
WFG	858.35	ESI (+) ionization	858.35→178.08	55
WFT	874.32	ESI (+) ionization	874.32→846.25	24
WFD	884.37	ESI (+) ionization	884.37→856.25	55
WFR	868.41	ESI (+) ionization	868.41→178.03	23
IS (2)	429.31	ESI (+) ionization	429.31→207.00	23

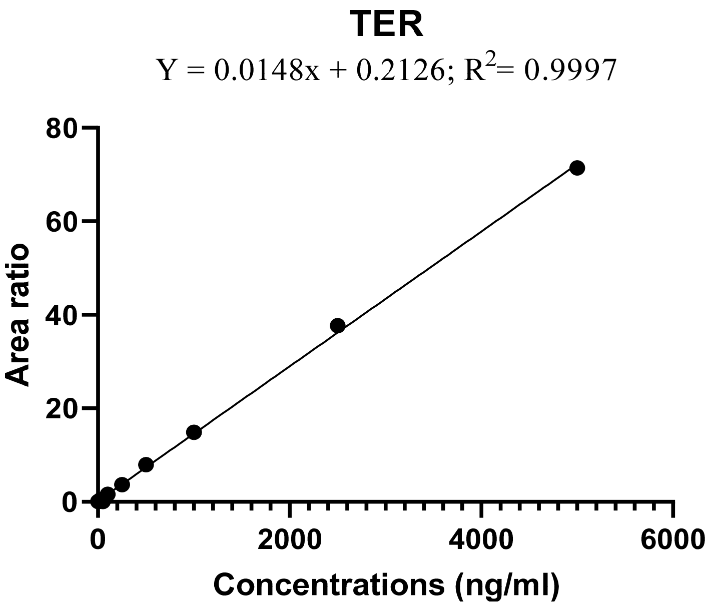

Figure S1. Linearity of TER in rats plasma samples.

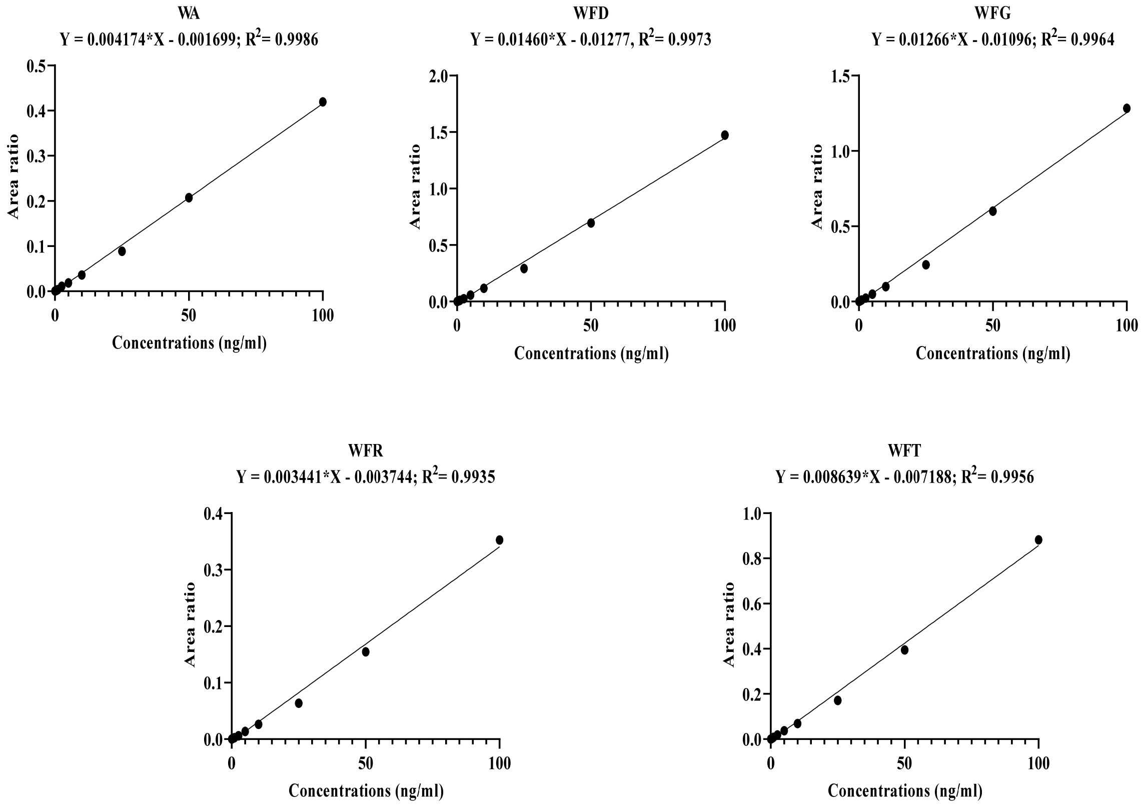

Figure S2. Linearity of WA, WFD, WFR, WFG, and WFT in rats plasma samples.
